# Reachability Does Not Explain the Middle Preference: A Comment on Bar-Hillel (2015)

**DOI:** 10.1177/2041669516639959

**Published:** 2016-03-28

**Authors:** Paul Rodway, Astrid Schepman, Volker Thoma

**Affiliations:** Department of Psychology, University of Chester, Chester, UK; Department of Psychology, University of Chester, Chester, UK; School of Psychology, University of East London, Water Lane, London, UK

**Keywords:** reaching/grasping, valence, middle bias, edge aversion, reachability, center-stage, location, choice

## Abstract

Choosing an object from an array of similar objects is a task that people complete frequently throughout their lives (e.g., choosing a can of soup from many cans of soup). Research has also demonstrated that items in the middle of an array or scene are looked at more often and are more likely to be chosen. This middle preference is surprisingly robust and widespread, having been found in a wide range of perceptual-motor tasks. In a recent review of the literature, Bar-Hillel (2015) proposes, among other things, that the middle preference is largely explained by the middle item being easier to reach, either physically or mentally. We specifically evaluate Bar-Hillel’s reachability explanation for choice in non-interactive situations in light of evidence showing an effect of item valence on such choices. This leads us to conclude that the center-stage heuristic account is a more plausible explanation of the middle preference.

## Introduction

A preference for items in the middle has consequences in many settings, including consumer choices (Atalay, Bodur, & Rasolofoarison, 2012), the assessment of people (Raghubir & Valenzuela, 2006), and responding to questionnaires (Bar-Hillel, 2015). Several explanations of the middle preference have been proposed, including the idea that it is a product of the central gaze bias (see Tatler, 2007), so that increased looking at central items increases their selection (Atalay et al., 2012), or because selecting the middle item somehow requires the least mental effort (Christenfeld, 1995). Another explanation is that people apply a heuristic that specifies that the best items are in the middle, and this influences their choice when there is little other information to discriminate between the items (Valenzuela & Raghubir, 2009).

A further explanation of the middle preference has recently been proposed by Bar-Hillel (2015). Her review of the literature aims to solve apparently contradictory effects of location on choice, for both serial choice tasks (where all options are presented one after the other) and simultaneous choice tasks (where all options are at view at the same time), by using a small number of psychological principles. Bar-Hillel has to be commended for her extensive work in this area (e.g., Bar-Hillel, 2011) and on her attempt to provide a parsimonious explanation of the many findings in this growing body of research. Unfortunately, however, we believe that the explanation she provides for the middle preference, specifically when the items to choose from are presented simultaneously rather than serially, is not compatible with a range of empirical data. For this reason, we felt compelled to redress the balance and place alternative interpretations center-stage.

Bar-Hillel (p. 431) suggests that the middle preference for simultaneously presented items is caused by up to three psychological processes: “(a) middle positions are more reachable in perceptual-motor tasks, (b) they are more representative in mental choice tasks, and (c) they are felt to be better hiding places in games of hide-and-seek.” Reachability in this sense applies not only just the physical ease with which an item can be held (e.g., proximity and time) but also the mental ease with which it comes to mind. Tasks where there might be an element of “hide-and-seek” between the person who set the task and the respondent include multiple-choice tests or interactive situations in which a participant wants to hide an object from a competitor (e.g., Attali & Bar-Hillel, 2003; Falk, Falk, & Ayton, 2009). Our primary concern is with the explanation provided by Bar-Hillel for the large number of studies showing a middle preference and which have not involved any element of hide-and-seek or differences in representativeness. For these studies, some of which were not included in her review (e.g., Kreplin, Thoma, & Rodway, 2014; Li & Epley, 2009; Reutskaja, Nagel, Camerer, & Rangel, 2011; Rodway, Schepman, & Lambert, 2012), the reachability explanation does not appear to be compatible with the data.

Bar-Hillel accepts that there are some findings she cannot explain in terms of reachability, such as the preference for the middle cubicles in a row of four toilet cubicles, when the left cubicle would have been the easiest to reach (Christenfeld, 1995). Other findings that do not appear explainable by reachability include the preference for the middle pen of three equidistant marker pens (Shaw, Bergen, Brown, & Gallagher, 2000). The pens were presented in a plastic case 6″ wide and taped to a wall. If we assume the majority of the 109 participants were right-handed any difference in reachability would be very slight but it would be in favor of the pen on the right, given the width of a person’s shoulders and the position of their hand. Bar-Hillel also asserts that the middle is easiest to reach because it reduces the likelihood of missing the pen compared to a pen on the edge. Contrary to Bar-Hillel’s assertion, it is possible that it is more difficult, physically, to take the middle pen due to the proximity of the two surrounding pens (see also Jackson, Jackson, & Rosicky, 1995), particularly if a person has large hands.

Reachability also does not appear to have played any role when selecting from items on a screen or paper, including the preference found for the person in the middle of a photograph of job candidates (Valenzuela & Raghubir, 2009), for artworks on a computer screen (Kreplin et al., 2014), for pictures on paper (Rodway et al., 2012), for consumer items online (Atalay et al., 2012; Reutskaja et al., 2011), when putting an “x” in one of three, or four, circles (Christenfeld, 1995), and when checking a box that matched the layout of a planogram (Atalay et al., 2012). For example, in Rodway et al.’s task, which involved selecting one of five pictures arranged in a row, if there was any influence of reachability then the rightmost picture might have been the easiest to choose given that all the participants were right-handed. Moreover, in Reutskaja et al.’s (2011) study, when nine consumer items were displayed in a 3 × 3 array on a computer screen, reachability does not seem to explain why the item in the middle was 60% more likely to be chosen than similar items at other locations. Finally, Atalay et al. presented products and planograms off-center, to the left or right of the participant, but this did not influence the middle preference, showing that it occurs even when the middle item is not the easiest to reach.

The other explanation of the middle preference, for which there is substantial evidence, is the center-stage heuristic (Raghubir & Valenzuela, 2006). This is a decision heuristic originating from the metacognitive belief that the best and most popular items are positioned in the center, and which underlies the preference for middle options in a wide range of circumstances (Valenzuela & Raghubir, 2009). Bar-Hillel states that she takes no issue with this account or evidence, but suggests that reachability is a better explanation because it is more parsimonious and makes clearer predictions. However, a crucial prediction of the heuristic that the “best items are in the middle” is that it might no longer apply when all of the items to choose from are clearly “bad” to the chooser (see Kreplin et al., 2014). Experimental evidence convincingly supports this prediction.

The importance of the valence of the items for eliciting a middle preference was first demonstrated in the data of Li and Epley (2009), although it was not analyzed in their study. We performed this analysis to further test the idea against data. When participants were asked to choose a face or painting from three simultaneously displayed good faces or good paintings, there was a significant preference for choosing the middle item (pooled choice numbers left to right: 26, 44, 27, χ^2^_(2)_ = 6.33, *p* = .04). In contrast, for three simultaneously displayed bad faces or bad paintings, there was no middle preference, but a significant right preference (pooled choices: 23, 28, 45, χ^2^_(2)_ = 8.313, *p* = .02; our conversion from percentages to numbers; our pooling and our analysis, based on data in Li and Epley, 2009, Table 1; studies 2(a) and 2(b), simultaneous). The center-stage heuristic, but not reachability, would predict the middle preference for the good items and not the bad items. Similarly, Kreplin et al. (2014) only found a middle preference when the items (artworks) had a positive valence and not a negative valence. Furthermore, an influence of valence was obtained by Rodway et al. (2012), but in this case it was the valence of the decision that differed rather than the items, with participants asked either “which item do you prefer?” or “which item do you least prefer?” The middle preference was only found for the “prefer?” decision and not the “least prefer” decision. This suggests that when people are asked to consider the item they least like, the center-stage heuristic, which specifies the location of the best item, no longer applies and the middle preference is eliminated. In contrast to the center-stage heuristic, it is not apparent how physical or mental reachability, or other explanations such as visual salience, could explain or predict these results.

We suggest that the results of Li and Epley (2009), and other studies showing a role of item valence (e.g., Kreplin et al., 2014), seriously question the validity of the reachability account. However, the center-stage heuristic may also require further specification, as different processes might be involved, such as the flanking items influencing the attractiveness of the middle item (Rodway, Schepman, & Lambert, 2013), or a negative emotional code being applied to items on the edge (see Scholtes, Dittrich, & Klauer, 2014). In addition, further experiments to directly test the reachability and center-stage accounts can be conducted. For instance, Bar-Hillel (p. 430) says that if Valenzuela and Raghubir’s (2009) participants had given a verbal response, rather than a motor response, then she would predict a preference for the edge rather than a middle preference, but this critical experiment has not been carried out. As suggested by a reviewer, it is also theoretically possible that in the majority of studies physical reachability (with right-handers tending toward the right item) and mental reachability (with western literate populations typically scanning from left to right) have operated in opposing directions, and caused the middle item to be chosen as a compromise. Although eye-tracking studies have shown that participants look first at the middle item (Atalay et al., 2012; Kreplin et al., 2014; see Tatler, 2007), this possibility could be tested further either by using a cross-cultural sample, who typically read from left to right, or with a population of left-handers. If a combination of physical and mental reachability causes the middle preference, then a preference for the left item, rather than the middle, would be predicted for left-handers.

We greatly admire and respect the detailed work that Bar-Hillel has completed on the cause of location-based choice and welcome further debate on this issue. However, we believe that Bar-Hillel’s explanation of the middle preference for simultaneously presented options is not supported by the evidence. An explanation in terms of the “middle-is best” heuristic is still persuasive and is better than the rest.
